# Quantitative 3D Characterization of Functionally Relevant Parameters in Heavy-Oxide-Supported 4d Metal Nanocatalysts

**DOI:** 10.1021/acs.chemmater.3c01163

**Published:** 2023-09-14

**Authors:** José Marqueses-Rodríguez, Ramón Manzorro, Justyna Grzonka, Antonio Jesús Jiménez-Benítez, Lionel Cervera Gontard, Ana Belén Hungría, José Juan Calvino, Miguel López-Haro

**Affiliations:** † Departamento de Ciencias de los Materiales e Ingeniería Metalúrgica y Química Inorgánica, Facultad de Ciencias, Universidad de Cádiz, Campus Rio San Pedro S/Nl, Puerto Real, 11510 Cádiz, Spain; †† Departamento de Física de la Materia Condensada, Facultad de Ciencias, Universidad de Cádiz, Campus Rio San Pedro S/Nl, Puerto Real, 11510 Cádiz, Spain

## Abstract

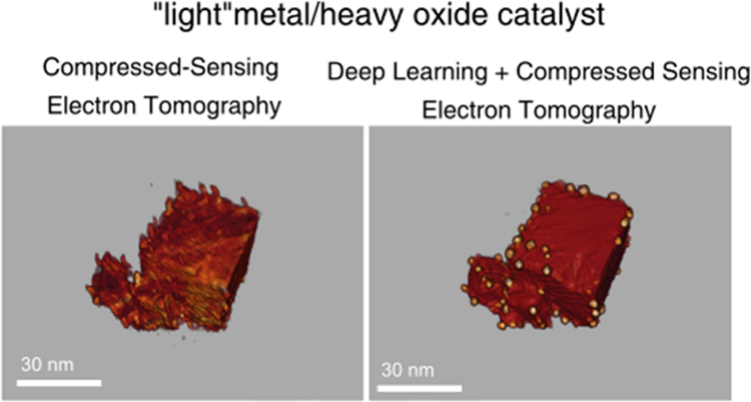

Accurate 3D nanometrology of catalysts with small nanometer-sized particles of light 3d or 4d metals supported on high-atomic-number oxides is crucial for understanding their functionality. However, performing quantitative 3D electron tomography analysis on systems involving metals like Pd, Ru, or Rh supported on heavy oxides (e.g., CeO_2_) poses significant challenges. The low atomic number (*Z*) of the metal complicates discrimination, especially for very small nanoparticles (1–3 nm). Conventional reconstruction methods successful for catalysts with 5d metals (e.g., Au, Pt, or Ir) fail to detect 4d metal particles in electron tomography reconstructions, as their contrasts cannot be effectively separated from those of the underlying support crystallites. To address this complex 3D characterization challenge, we have developed a full deep learning (DL) pipeline that combines multiple neural networks, each one optimized for a specific image-processing task. In particular, single-image super-resolution (SR) techniques are used to intelligently denoise and enhance the quality of the tomographic tilt series. U-net generative adversarial network algorithms are employed for image restoration and correcting alignment-related artifacts in the tilt series. Finally, semantic segmentation, utilizing a U-net-based convolutional neural network, splits the 3D volumes into their components (metal and support). This approach enables the visualization of subnanometer-sized 4d metal particles and allows for the quantitative extraction of catalytically relevant structural information, such as particle size, sphericity, and truncation, from compressed sensing electron tomography volume reconstructions. We demonstrate the potential of this approach by characterizing nanoparticles of a metal widely used in catalysis, Pd (*Z* = 46), supported on CeO_2_, a very high density (7.22 g/cm^3^) oxide involving a quite high-atomic-number element, Ce (*Z* = 58).

## Introduction

Electron tomography (ET) is a well-established technique to characterize nanomaterials in three dimensions (3D) both at nanometer and atomic scales.^1,2^ In particular, in the field of heterogeneous and environmental catalysis, ET in high-angle annular dark-field (HAADF) scanning transmission electron microscopy (STEM) mode has played an important role not only in understanding the behavior of the catalytic materials but also in designing new catalysts with more active phases.^3^

HAADF-STEM ET has mainly been used to study catalysts composed of unsupported or low-atomic-number substrate (e.g., carbon, alumina, silica)-supported nanoparticles, providing detailed information on their morphology, location, and spatial distribution.^4−8^ However, there is a scarcity of studies involving supports of high-atomic-number elements like lanthanide oxides. Specifically, there are limited studies on ceria-based oxides, which have significant applications in catalysis, especially in environmental protection and H_2_ production.^9^ Existing electron tomography studies have mainly focused on characterizing the 3D properties of pure or mixed ceria oxide nanocrystals, emphasizing morphological features and spatial distribution of electronic states, such as surface and bulk Ce^3+^/Ce^4+^ contents.^10−14^

ET studies involving ceria oxides as supports for metallic phases are even more limited despite the widespread application of these materials. This limitation is likely due to the inherent complexity of these systems, which can be attributed to two factors. First, the catalysts typically have low metallic loadings, resulting in a low surface density of particles that are generally very small (<5 nm). Second, there is a small atomic number difference, Δ*Z*, between the metallic phase and Ce, making the detection of the former extremely challenging, especially for particles in the very small size range.

Previous research conducted in our laboratory utilized HAADF-STEM tomography to investigate the 3D spatial distribution of gold (Au) nanoparticles on Ce–Tb–Zr mixed oxides. In that study, the reconstruction of the 3D structure was performed using conventional algorithms, and the separation of contrast between the metal and support was carried out manually.^15^ This so-called segmentation step, which involves distinguishing the intensities corresponding to each component in the reconstructed volume, is crucial for quantifying structural properties.

More recently, a methodology combining advanced image-processing algorithms to denoise each of the images comprising the whole set of projections acquired in the tomographic experiment (tilt series) and compressed sensing-based (CS) algorithms for more reliable 3D signal recovery enabled a quantitative 3D analysis of Au catalysts supported on CeO_2_. Using this approach, the volume representing the metal nanoparticles could be accurately segmented from the support volume using a semiautomated procedure. Various properties of the metal phase (average nanoparticle size and metal dispersion), the support (specific surface area), and the overall system (metal loading) were estimated from the segmented volumes. These estimates exhibited excellent agreement with values obtained through macroscopic techniques such as N_2_ physisorption isotherms, X-ray fluorescence, and 2D STEM microscopy-based metal particle size distributions. Bouziane et al. further advanced the tomographic analysis of this type of catalyst by quantitatively evaluating the expected accuracy of these measurements using material-realistic 3D models.^16^

The higher value of the atomic number of Au (*Z* = 79) with respect to Ce (*Z* = 58) allows for easy differentiation of Au nanoparticles from CeO_2_ in HAADF-STEM images. However, for catalysts based on lighter metals like Pd (*Z* = 46), visualizing small Pd nanoparticles supported on CeO_2_, even in 2D HAADF-STEM projections, is challenging.^17−19^ In fact, it has been demonstrated that detecting Ru and Pd nanoparticles on Ce–Zr mixed oxides using HAADF-STEM is difficult, with better visibility when they are imaged in the top view.^18,20^ Effectively, in the top view projection, high-angle scattered electrons experience secondary scattering in the support, creating contrast. However, when imaged in the profile view, the contrast between the metal and support is solely determined by the difference in atomic number (Δ*Z*) and relative size. Due to the larger support crystallite size and lower metal atomic number compared to Ce, the intensity of metal nanoparticles is much lower than that of the support, making their detection difficult in the profile view. Consequently, in HAADF-STEM ET experiments with 3d or 4d catalysts on cerium or cerium-based oxides, nanoparticles are visible only in certain projections.

This loss of information leads to inaccurate reconstructions when using classical algebraic algorithms, like SIRT (simultaneous iterative reconstruction technique) or WBP (weighted back projection). Additionally, the presence of noise, common in experimental HAADF-STEM images, further complicates the reconstruction process.

Compressed sensing algorithms, particularly those based on total variation minimization (TVM), have demonstrated as highly efficient in addressing the information loss in such materials, enhancing 3D analysis of catalysts, even starting from tilt series with a limited number of projections.^21^ However, as proven in this study, relying solely on these robust algorithms demonstrates insufficiency to successfully overcome the complex 3D characterization challenges posed by 4d metals supported on ceria oxides.

Recent advancements in deep learning (DL) methodologies have proven highly effective in improving the quality of electron tomography (ET) studies.^22^ For example, DL techniques have been successfully employed to reconstruct 3D structures of systems like ZnSe@ZnS quantum dots involving components with certain compositional proximity.^23^ This technique has also been utilized to dump the effects of the missing wedge and enhance resolution in ET, as demonstrated by Wang et al.^24^ Furthermore, these methods have also been applied to denoise energy-dispersive X-ray (EDX) elemental maps and improve analytical electron tomography studies.^25,26^ Recently, DL algorithms have also been successfully used for automatic segmentation in a large data set of ET experiments involving Pt nanoparticles supported on alumina.^27^

Based on these recent findings, we hypothesize that DL methods can improve the analysis of the limited contrast differences observed in HAADF-STEM images of nanocatalysts with 3d-4d metals supported on ceria oxide particles. It is expected that the proven capabilities of DL for denoising images, dumping reconstruction artifacts, and improving segmentation open a route to a reliable 3D nanometrology of this type of catalysts. In particular, this work explores the potential of combining compressed sensing and deep learning techniques to reach this goal.

**Figure 1:**
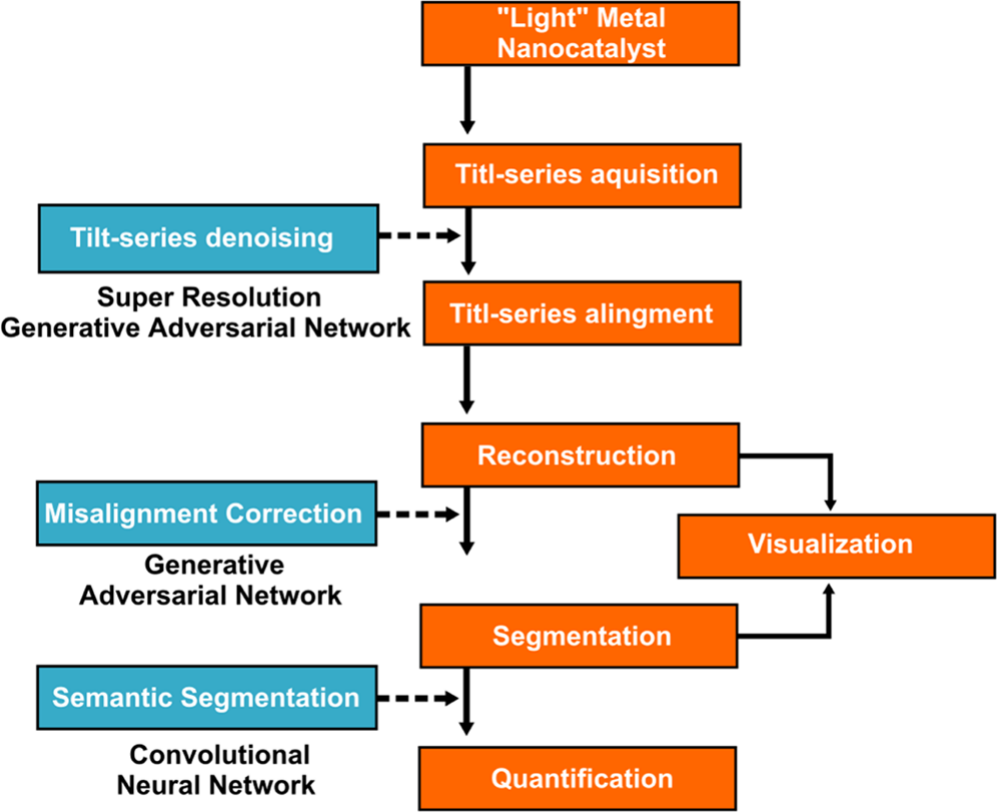
Principal steps in electron tomography.

As depicted in Figure 1, 3D nanometrology involves a number of consecutive steps, going from the acquisition of the experimental tilt series to the quantification of properties from the reconstructions. Therefore, to improve the whole process, a DL-based pipeline incorporating different neural networks is required. Each neural network must be optimized for a specific image-processing task. Thus, two different types of generative adversarial network (GAN) algorithms were fine-tuned to enhance contrasts in the experimental HAADF-STEM images and correct misalignments in the reconstructed volume. Likewise, a convolutional neural network (CNN) was optimized for semantic segmentation of the reconstructed volumes.

The implemented deep learning methods were the supervised ones and required previous training. Therefore, to perform this task, a set of perfectly known starting data, which are considered the “ground truth”, is necessary. A large fraction of the data in this set is used to tune the parameters in the DL algorithm so as to provide the correct answers. The remaining part of the training set is then used to check that, after training, the responses provided by the algorithm are, in fact, correct. This last step is called validation. After training and validation, the algorithm would be prepared to produce a satisfactory output from a problem case not included in the training set. As expected, the quality of the training depends both on the size and completeness of the training data set and the closeness between the examples included in the training set and the problem.

In our case, we are dealing with HAADF-STEM images. Therefore, computer scripts were developed to automatically build a large set of synthetic models with structural features closely resembling those observed in experimental images. The use of randomly generated, material-realistic, 3D synthetic models constitutes another innovative aspect of our approach.

## Results and Discussion

**Figure 2:**
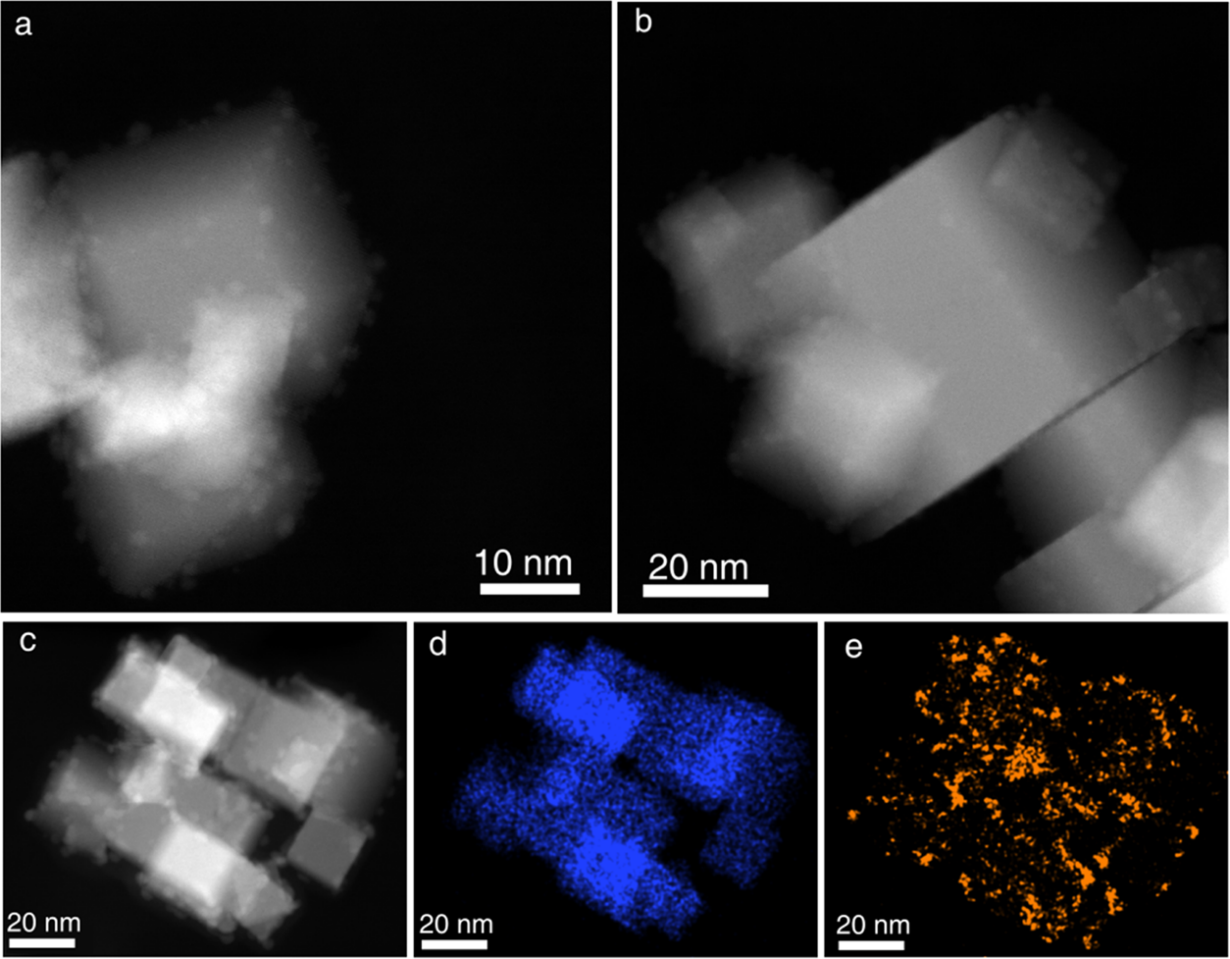
(a–c) Representative experimental HAADF-STEM images of Pd nanoparticles supported on CeO_2_ nanocubes and (c–e) elemental X-ray mapping of a Pd/CeO_2_ crystallite bunch containing a large number of catalyst particles. The Ce–L signal is shown in blue and in orange, corresponding to the Pd–L signal.

Figure 2a,b shows two representative experimental, medium magnification, HAADF-STEM images of a Pd nanocatalyst supported on CeO_2_ nanocubes. To properly understand the contrasts in these images, the following factors need to be considered: (1) the location of nanoparticles with respect to the support in the projection; (2) the relative dimensions of the two components in the system; and (3) the parameters governing image intensity in HAADF imaging.

Regarding the first, it is important to clarify that the metal nanoparticles can be observed either in top or planar view conditions, *i.e.*, on a support background, or in a so-called profile view, *i.e.*, with a vacuum background. The latter corresponds to those particles located, in projection, on the edge of the support crystallites.

Regarding the second question, two aspects are important, first that the dimensions of the support crystallites will, in general, be at least one order of magnitude larger than that of the metal entities, particularly at those sites where several catalyst particles overlap in projection, which is quite frequent due to usual aggregation of these particles into bunches.

Finally, regarding the third factor, simply recall that HAADF-STEM image intensity depends both on roughly the squared atomic number of the elements in the atomic column (*I*∝*Z*^2^) and the length of the column (*i.e.*, local thickness). In this respect, it is important to take into account the atomic numbers of Ce (*Z* = 58) and Pd (*Z* = 46).

Note that both in the locations corresponding to thick areas of large cubes or those where several smaller cubes overlap in projection (Figure 2a,b), image contrasts hardly allow the detection of small Pd nanoparticles. In these sites, the presence of Pd is only revealed in EDX maps, as shown in Figure 2c–e. As expected, the large contribution of the support to the overall image intensity severely dumps that of the small metal particles. In other words, the mass-thickness contribution of the support hampers the visibility of Pd nanoparticles.

In contrast, in areas with non-overlapping or small cubes (Figure 2b), the larger relative contribution of metal nanoparticles to overall intensity allows the detection of Pd nanoparticles. Particularly, particles in the top view exhibit slightly higher intensities than the surrounding support areas. This observation is consistent with previous studies on Ru particles supported on Ce–Zr mixed oxides,^20^ where the visibility of nanoparticles in the top view is attributed to consecutive scattering of electrons. Electrons scattered at high angles by the metal nanoparticle undergo additional scattering by the support crystallite. As a result, the total number of electrons scattered in the metal areas is greater than in neighboring, metal-free areas, making them more easily detectable.

**Figure 3:**
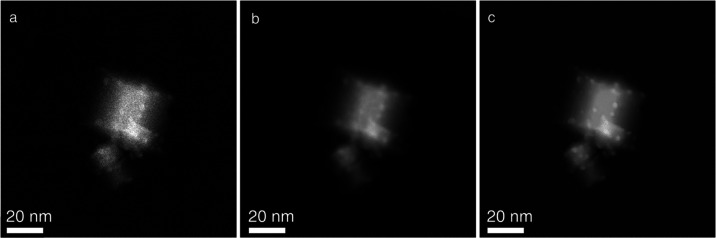
(a) Experimental HAADF-STEM image of a small bunch of Pd/CeO_2_ catalyst particles; (b) image of the same aggregate after denoising using a combination of Anscombe and undecimated wavelet transforms (AT-UWT); and (c) image after denoising by ESRGAN.

This contrast mechanism remains effective as long as the contribution due to the metal nanoparticle holds significant and above image background fluctuations. Therefore, for visualizing “light” metal nanoparticles supported on heavy oxides in HAADF-STEM images, isolated and small cubes are preferred, as shown in the HAADF-STEM image in Figure 3. This shows an inhomogeneous distribution of metal nanoparticles, with sizes ranging from 1 to 4 nm, onto the ceria support crystallites.

Several studies have reported that the use of smart algorithms to denoise tilt series provides an improvement in the final reconstructions.^28,29^ More recently, advanced algorithms based on deep learning methods have also been employed to denoise analytical electron tomography tilt series.^25^ In particular, using convolutional neural network architectures, the volume of core–shell structures has been reconstructed. Likewise, single-image super-resolution (SR) techniques based on deep learning have also proven to be effective in improving image quality. Specifically, SR techniques using generative adversarial networks (GANs) have been used to enhance low-quality digital images by not only reducing noise but also improving sharpness and contrast. These last features are, in fact, key for reconstructing a tilt series of ″light″ metals supported on heavy oxides. While this approach has been used to improve image resolution in scanning electron microscopy focused ion beam (SEM FIB)-3D data,^30^ to the best of our knowledge, it has not yet been used with HAADF-STEM images to study complex nanomaterials such as Pd nanocatalysts supported on heavy oxides. Therefore, to improve our experimental data set, we have modified an existing ESRGAN (enhanced super-resolution generative adversarial network).^31^ This is a compulsory step to better adapt the characteristics of this type of network architecture to HAADF-STEM images.

GAN models are based on the so-called adversarial learning. This learning paradigm has two main parts, both of which are neural networks: the discriminator (D) and the generator (G). The discriminator takes in the input data and determines whether it is fake or real, while the generator generates fake data to try to fool the discriminator. Thus, the network must learn to distinguish between fake and true data. To make the learning process robust, both the discriminator and the generator architectures must be trained.

As previously mentioned, the performance of DL models is limited by the quality and volume of the training data set. So, a homemade script was created to generate synthetic models resembling experimental Pd/CeO_2_ HAADF-STEM images. These synthetic models include features like nanoparticle and support intensity, noise, etc. Aiming to closely mimic the morphological characteristics of supported metal catalysts, they comprised aggregates of randomly oriented prism-shaped crystallites as support structures. Smaller truncated-sphere-shaped objects representing metal particles were then distributed onto these prismatic crystallites. The number, diameter, truncation degree, and precise positions of these metal particles on the support surface were also randomly selected. A total of 100 different synthetic models were employed.

To reproduce the relative intensities observed in the experimental HAADF-STEM images, the product of density and thickness (i.e., *I*_HAADF_ α ρt) and the density of CeO_2_ and Pd were taken into account. Subsequently, the models were projected from −70° to +70 every 5°, and a mixture of Poisson and Gaussian noise was added, resulting in a total of 1500 synthetic images. Then, 80% of these images were used for training, and the remaining 20% were used to validate our DL model. Some representative models used to train and validate the DL model are shown in Figure S1.

To estimate, in quantitative terms, the improvement in denoising the experimental images by using ESRGAN, the peak signal-to-noise ratio (PSNR)^32^ and structural similarity index measure (SSIM)^33^ have been estimated for a projected synthetic model with varying levels of noise; Figure SI 2. The results are compared with those obtained in the presence of noise and after denoising using alternative techniques. In particular, data corresponding to a combination of Anscombe and undecimated wavelet transforms (AT-UWT) and RIDnet, a convolutional neural network, are shown. It is noteworthy that denoising carried out by ESRGAN provides outstanding values for both PSNR and SSIM, which clearly improve the quality of the recorded tilt series.

Figure 3 illustrates the application of the ESRGAN DL denoising to an image of an experimental HAADF-STEM electron tomography tilt series. In particular, the DL-denoised image (Figure 3c) is compared with both the raw image (Figure 3a) and the one obtained using a combination of Anscombe and undecimated wavelet transform (AT-UWT) denoising (Figure 3b). The AT-UWT denoised image was obtained using the *J* = 1 scale, as reported in ref (29).

**Figure 4:**
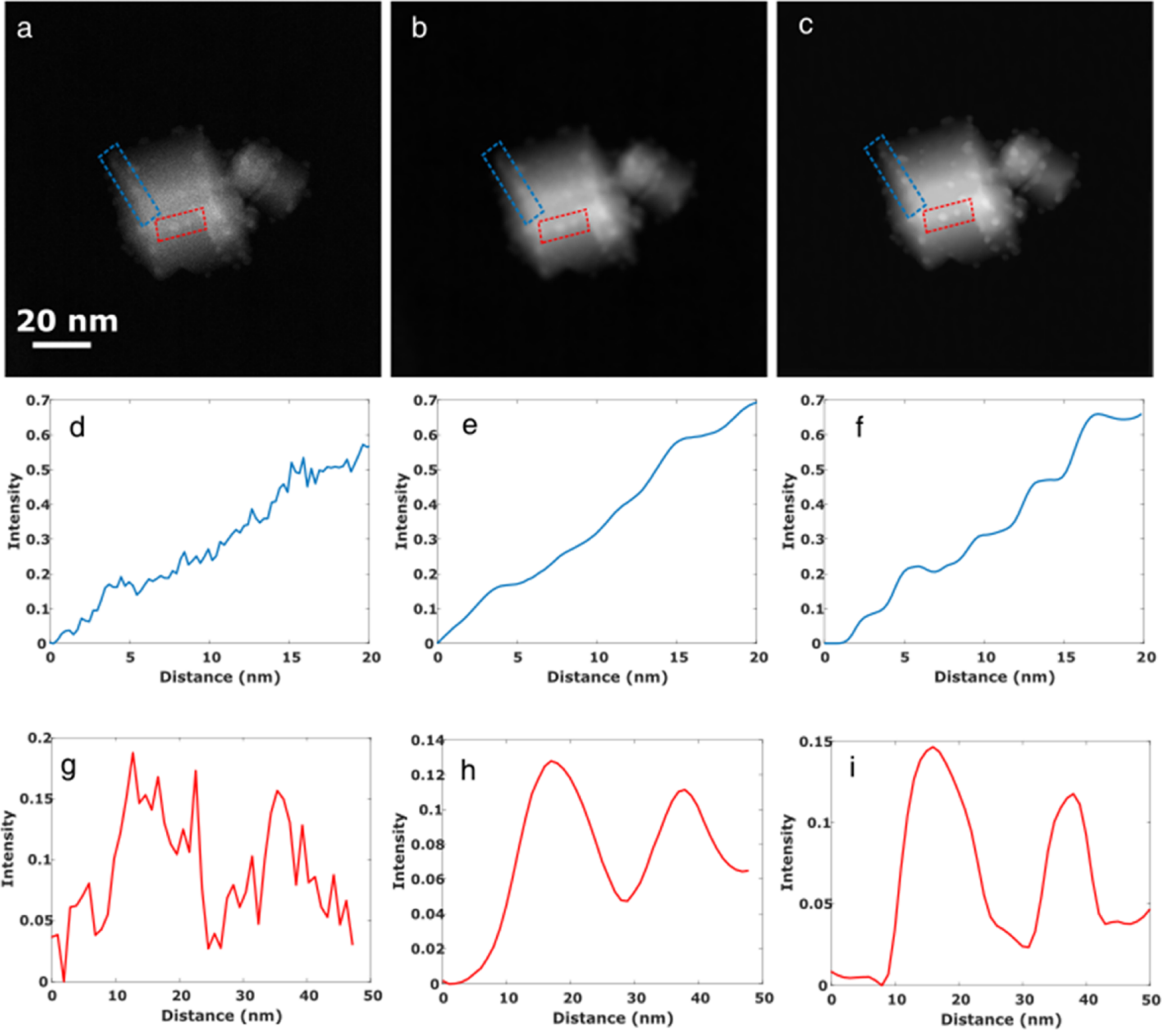
Comparison between raw (a), AT-UWT (b), and deep learning (c) denoised images; intensity profile along particles imaged in the profile view (blue box) in the raw (d), AT-UWT (e), and DL (f) denoised images; (g–i) same as panel (b) but with particles observed in top view conditions (red box). Note how, in both cases (profile and top view conditions), there is a remarkable improvement in the contrasts associated with the small metal particles.

To assess the quality of our experimental images, image intensity line profiles along particles were recorded both in profile (blue box) and top view (red box) conditions, on the raw HAADF-STEM (Figure 4d,g), AT-UWT (Figure 4e,h), and DL-denoised (Figure 4f,i) images. A comparison of the different profiles proves a clear improvement of the original signal after DL denoising. Moreover, though both denoising techniques improve the recovery of the intensity of the nanoparticles, the peaks in the DL method are clearly sharper than those from AT-UWT, which is in agreement with the results obtained from a direct, naked-eye inspection of the images.

The enhancement is especially significant for small particles located in the profile view (blue box). Therefore, our DL approach not only removes noise but also enhances the signal-to-noise ratio. The improvements obtained in denoising, sharpness, and signal-to-noise ratio are consistent with previous results obtained using SR DL techniques to enhance digital images. This is particularly relevant for visualizing the nanoparticles in the corresponding tomogram and then extracting relevant information to better understand the behavior of nanocatalysts.

The HAADF-STEM tilt series denoised using DL methods were aligned using a marker-less technique. This involved two steps: (i) correcting the XY image shift between consecutive images using a cross-correlation algorithm and (ii) correcting the tilt axis rotation. The second step is more challenging to solve and can result in inaccurate reconstructions. In fact, any residual misalignment between the true and estimated rotation axis spreads the signal from the reconstructed object and produces “arcs” of intensity. The direction of the arc depends on the direction of the misalignment away from the correct axis, and the degree of spread is dependent on the magnitude of that misalignment. This so-called “arc” distortion affects more evidently small objects, modifying their morphology in the reconstruction.^34^ Despite this, this crucial stage is still routinely carried out manually, and very little has been explored to improve it, most likely due to the intrinsic difficulty of developing a methodology of general applicability using conventional image-restoration algorithms.

At this point, we decided to explore if DL methods could correct the distortions produced by small misalignments. Since GAN models have been demonstrated to produce more detailed and higher quality images than CNN algorithms,^35,36^ we developed a restoration method based on the first type of architecture. This GAN model was applied as a post-processing step of the reconstructed volumes. To avoid any additional distortion due to the missing wedge, the TVM algorithm was used for reconstructions. Also worth noting, the GAN model was modified using a U-net structure for the generator (G) (Figure SI 3), while the conventional architecture used in these models was kept for the discriminator (D). This alteration in the GAN model is commonly known as a U-net-based GAN, which is a type of conditional GAN.^36^ The advantage of this model relies on the fact that it allows one to take an image as the input instead of random noise.

The proposed GAN model is supervised and therefore both the G and the D components were properly trained. Additional details regarding this step can be found in the Supporting Information section. The robustness of the proposed methodology was validated by estimating the SSMI of reconstructed synthetic models after correcting tilt misalignment using the U-net GAN architecture. Figure SI 4 reveals that the proposed GAN model provides SSMI values quite close to 1, which is, in fact, a very remarkable fact. In other words, the results obtained from the synthetic models demonstrate that this methodology can be reliably applied as a post-processing step to enhance the quality of the final reconstructed volume.

**Figure 5:**
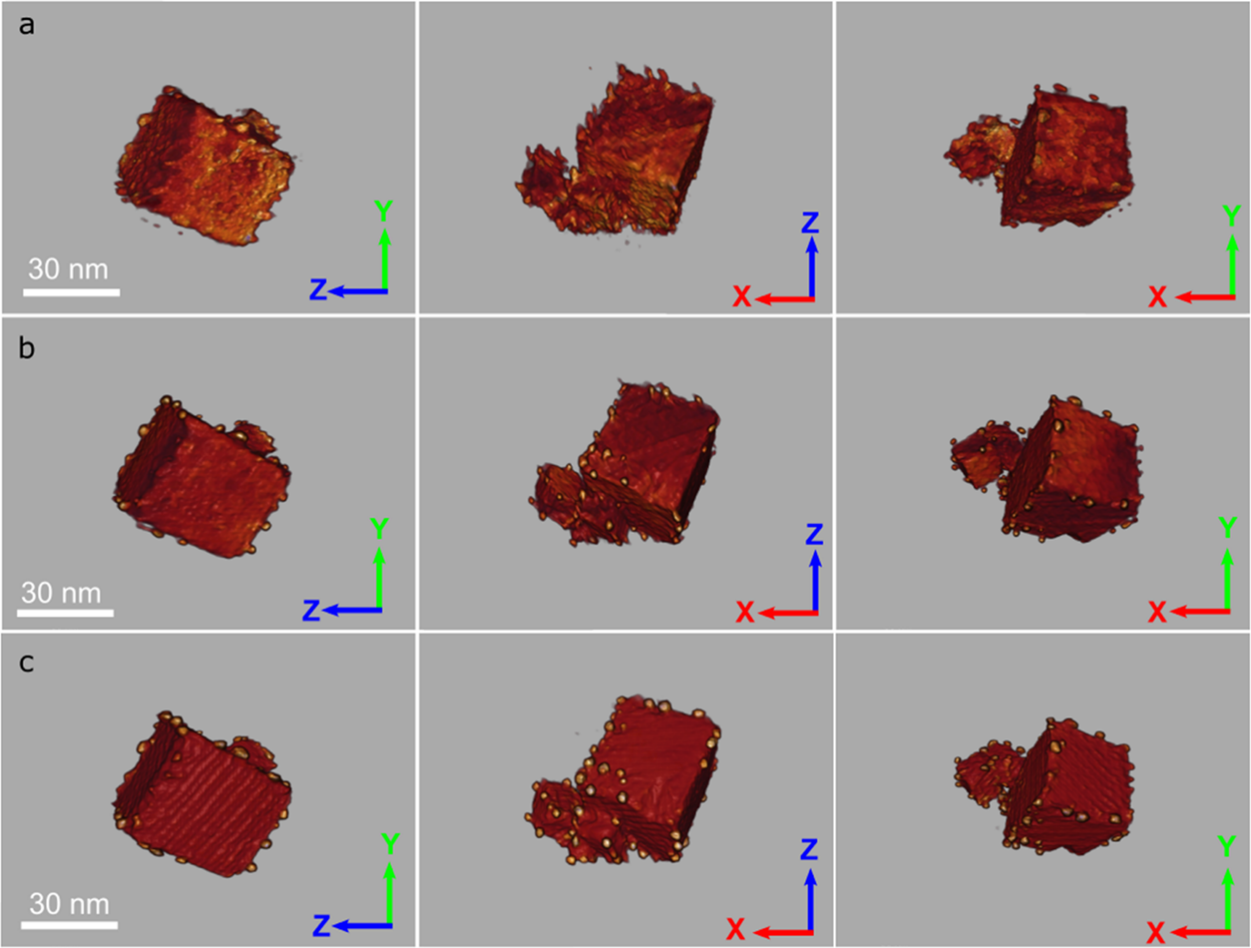
3D voxel representations, in three different perspective views, of the HAADF-STEM TVM raw reconstruction (a), HAADF-STEM DL-denoised TVM reconstruction (b), and HAADF-STEM DL-denoised TVM image-restoration reconstruction (c).

Figure 5 depicts three different perspective views of the 3D voxel representation of the reconstructions obtained from the same set of HAADF-STEM tilt series: TVM raw reconstruction (Figure 5a), DL-denoised+TVM reconstruction (Figure 5b), and DL-denoised+TVM+ DL image-restoration reconstruction (Figure 5c). The raw TVM reconstruction leads to a very noisy output, in which it is very difficult to distinguish the presence of Pd nanoparticles, as their intensities are similar to those of the support. Moreover, the Pd nanoparticles exhibit a distorted morphology due to the likely contribution of a small misalignment of the tilt axis, as shown in Figure 5a (middle column). Since it is well established that alignment quality is highly influenced by the contrast and noise of the tilt series images, this artifact is expected.

Figure 5b shows the 3D volume rendering of the reconstructed volume from the DL-denoised tilt series. Comparison with the reconstruction obtained from the raw images reveals a clear improvement in visualizing Pd nanoparticles. The ESRGAN algorithm enhances the signal-to-noise ratio, leading to a better-quality final volume reconstruction. However, some imperfections in nanoparticle morphology remain evident in the 3D voxel rendering viewed along the XZ orientation; Figure 5b (middle column). To address this limitation, the DL image-restoration approach (Figure 5c) was applied, resulting in a remarkable improvement and superior quality of the final volume reconstruction in all perspective views of the 3D voxel representation. The enhanced sharpness and absence of morphological distortions in Pd nanoparticles, as well as the improved morphology of the ceria support crystallites, allow for precise identification of the location of supported nanoparticles and their crystallographic interactions with the support.

In summary, the DL approach does not only allow for clearly detecting the Pd nanoparticles but also overcoming morphological distortions in both components of the system, metal, and support crystallites.

Winckelmans et al. have proposed that collecting electrons scattered at both medium (MAADF) and low angles (LAADF) can enhance the signal of elements with lower atomic numbers.^37^ They used this imaging mode to investigate the growth mechanism and internal structure of Au nanoparticles grown onto Pd seeds. Therefore, the potential of using these alternative imaging modes combined with the DL techniques was also considered. However, in our case, the LAADF-STEM images, which collect fewer incoherent electrons, lead to 3D reconstructions containing strong artifacts due to the contribution, in a significant number of images in the tilt series, of diffraction contrast effects. It has to be taken into account that the areas selected for reconstruction contain very often a bunch of crystallites in random orientations with respect to each other. This makes it, most of the time, impossible to select even one image in the tilt series where diffraction effects are absent simultaneously in all of the crystallites.

Regarding the reconstructions obtained using MAADF-STEM images, their quality was very close to that observed in HAADF-STEM reconstructions. However, since MAADF images can eventually contain a certain contribution of diffraction effects, HAADF-STEM-based reconstructions have become more reliable.

The results presented up to this point clearly demonstrate the need to employ the most advanced post-processing algorithms, such as those based on SR-deep learning techniques for denoising tilt series and conditional GANs for enhancing reconstructions based on compressed sensing. These tools allow us to face quite complex 3D characterization challenges, as that considered here in connection with small nanoparticles of a light, 4d, metal supported on a heavy oxide. In fact, this combination of advanced techniques is an unavoidable requirement to obtain high-fidelity reconstructions. As illustrated below, these reconstructions enable accurate and precise quantitative measurements of structural parameters, which could be further used to establish correlations with the functionality of such systems.

**Figure 6:**
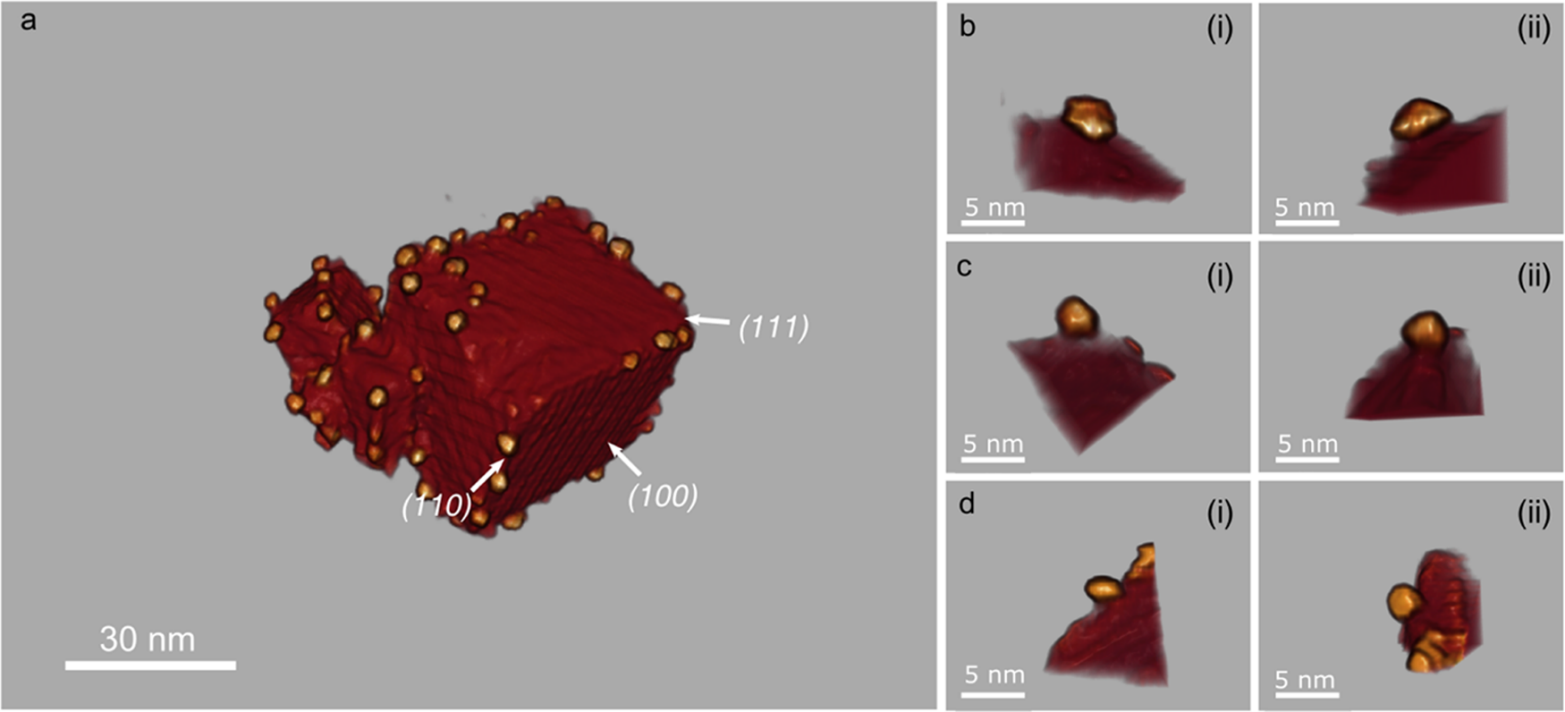
(a) 3D perspective view illustrating the spatial distribution of Pd nanoparticles supported on ceria cubes (a). (b–d) Three representative Pd nanoparticles extracted from the 3D volume render of the HAADF-STEM DL-denoised CS-ET image-restoration reconstruction in panel (a). For each particle, two different perspective views (i) and (ii) are shown.

In this sense, and to begin with, a qualitative analysis based on a direct visual inspection was conducted to study the morphology and spatial distribution of Pd nanocatalysts supported on ceria nanocubes. In this respect, Figure 6a indicates that the Pd nanoparticles are mainly located at the edges and corners of the nanocubes. These sites are associated, respectively, with {110} and {111} facets of the fluorite structure of the support, which make up roughly only 16% of the total exposed surface area of the nanocubes.^38^ The extended flat facets, which correspond to {100} surfaces, are mostly bare. The volume reconstruction clearly demonstrates that Pd nanoparticles have a marked preference for specific crystallographic surfaces when the deposition–precipitation (DP) method is used to prepare the catalysts. This feature is likely to have a significant effect on catalytic performance.

This singular behavior has also been observed in Au nanocatalysts supported on ceria nanocubes prepared by DP.^38^ Gold particles are located on {110} and {111} facets. Moreover, a preferential distribution on {111} surfaces and defects was observed in ET experiments performed on a Au/CeO_2_-ZrO_2_ low-surface area catalyst, where the contribution of this type of facet was higher.^15^

The preferential distribution of Pd onto the support crystallites does not depend on the size of the cubes. This can be appreciated in Figure 6a, where three cubes of different sizes are observed, and in all cases, Pd is located mostly on the edges. The same is observed in other reconstructions obtained from this catalyst, like the one shown in Figure SI 5.

Taking into account the Pd loading, the surface area of the catalyst (18 m^2^/g), the average particle size, the width of the {110} edges (around 2 nm), and the average dimensions of the nanocubes (45 nm), an average number of 5–7 Pd nanoparticles per cube edge could be roughly estimated. This figure is in the order of magnitude observed in the reconstructions, which, therefore, also agrees with a preferential distribution of Pd on the edges.

The growth of Pd nanoparticles occurs on the edges of nanocubes, specifically on {110} facets. Given that both components have *fcc* cubic lattices, it is plausible to assume contact between CeO_2_ and Pd {110} planes. Previous studies have demonstrated the epitaxial growth of Rh with a parallel orientation relationship onto {111} CeO_2_.^39^

A comparison of the nonpolar {110} planes of CeO_2_ (Figure SI 6a) with the {110} plane of Pd (Figure SI 6b) reveals that after a 1.8% contraction of the Pd lattice, a perfect 1 CeO_2_:2 Pd match could occur between the two structures when aligning the [001] CeO_2_ and [1-10] Pd directions. Furthermore, the Ce and O distribution within the {110} planes coincides with that observed in the PdO {110} planes, apart from dimensional factors.

The interaction with {110} crystallographic surfaces is expected to influence both the growth of the nanoparticles in terms of their size and their morphology in terms of truncation.^40^ In this respect, Figure 6b–d displays three representative Pd nanoparticles extracted from the 3D rendered volume. Note how the 3D reconstructions indicate that the nanoparticles are well-faceted, in good agreement with HR-HAADF and HR-BF STEM images acquired on the same catalysts, Figure SI 7. It is also clear that the Pd particles present different height/width aspect ratios, which must be related to the balance between bulk and interface energies.

To perform an accurate quantitative analysis of morphological parameters with statistical significance, the reconstruction was segmented. Segmentation involves separating the entire reconstructed volume into the background and objects of interest. This image-processing step is that in which deep learning technology has been most widely applied, especially in the context of computer vision. In this field, DL segmentation tries to provide an answer to the following questions: what is in this image, and where in the image is it located? To this end, methods based on so-called semantic segmentation have been used.

This approach generates a segmentation map in which the pixel values of the input image are transformed into class label values. Among the convolutional neural networks, those based on a U-net architecture have shown excellent performance in this task.^36^ Therefore, a U-net network was implemented to perform semantic segmentation and automatically separate the different components of the enhanced volume reconstructions, i.e., metal nanoparticles, support, and background. This algorithm is also supervised, so a training of the neural network was performed.

Following the approach previously employed to train the DL algorithm used to improve the experimental tilt series, a new database comprising 25 synthetic models of truncated spheres supported on cubes, which simulated the nanocatalyst, was built. This database also contained the models of the isolated truncated spheres and isolated cubes. The whole set (75 models in total) was projected from −90 to 90° every 5°. To reproduce the relative intensities of the two components (Pd and CeO_2_) in the experimental HAADF-STEM images, the product of density and thickness (i.e., *I*_HAADF_ α ρ*t*), together with the density values of ceria and palladium, were considered. Then, a mixture of a random amount of Poisson and Gaussian noise was added to each projected nanocatalyst model.

To mimic the experimental procedure used to reconstruct the experimental images, the noised projections were denoised using our ESRGAN approach. Afterward, the denoised projections were reconstructed by using a TVM algorithm. Note that for each nanocatalyst reconstructed model, its counterpart of isolated particles and cube models were also reconstructed, which makes a total of 75 3D reconstructions. The reconstructions of the isolated components were then used as the ground truth for the nanoparticles and support.

The training was performed slice-by-slice, so the final database constructed to validate our DL segmentation approach contained a total of 19200 images (256 × 25 × 3). From this database, 80% was used for training and validating (split into 64% for training and 16% for validating) and the remaining 20% for testing.

**Figure 7:**
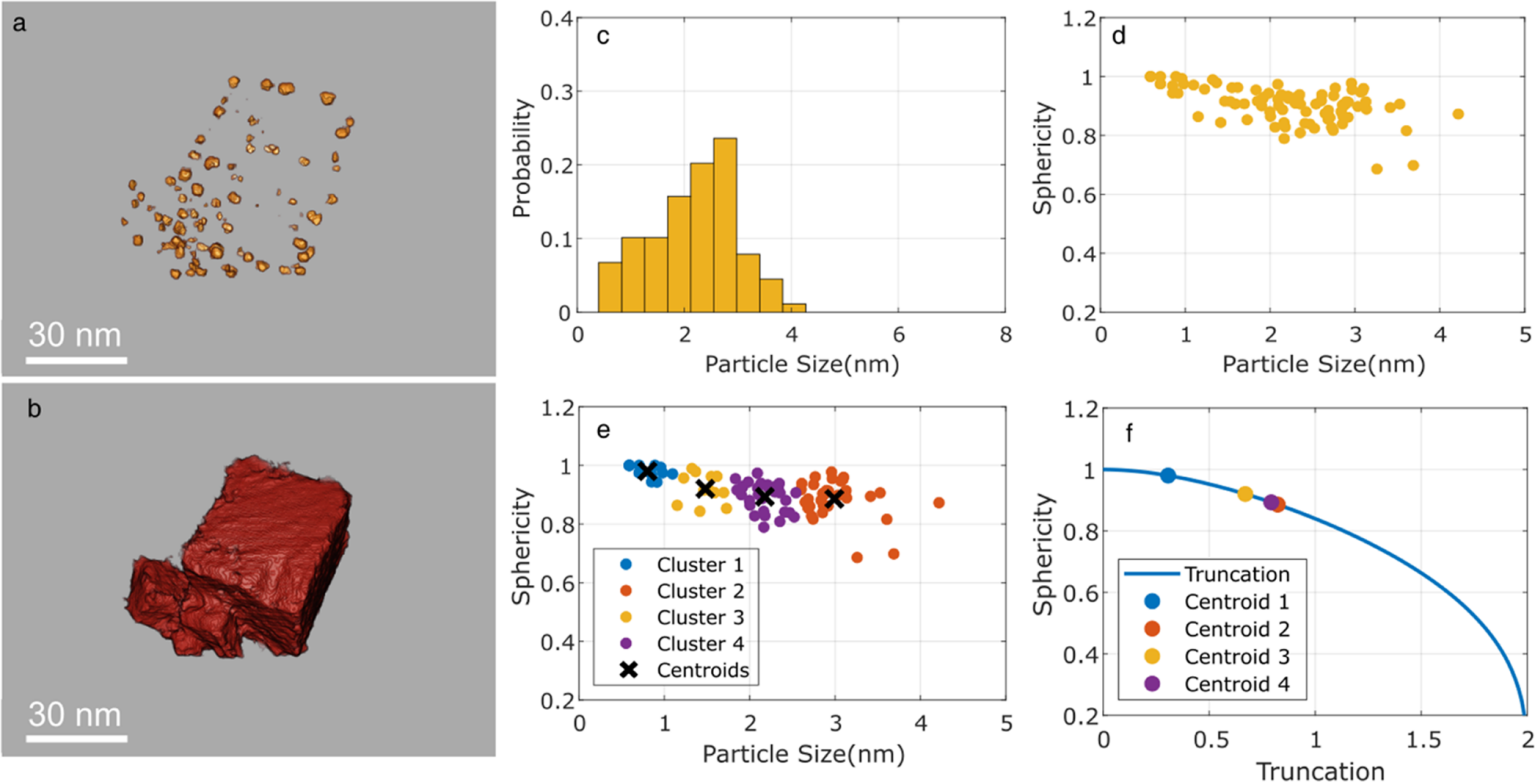
3D rendered surface after applying the semantic segmentation of Pd nanoparticles (a) and ceria support (b). Pd particle size distribution obtained from quantification of the volume segmentation (c). Sphericity (ε) of all of the segmented Pd particles as a function of size (d). Results of the K-means cluster analysis of Pd particle sphericity as a function of size (e). (f) Evaluation of particle truncation (α) as a function of sphericity.

Figure 7 displays the result obtained after applying the semantic segmentation methodology to the DL-denoised TVM image-restoration reconstruction of the experimental tilt series. It is remarkable how the Pd nanoparticles (Figure 7a) are neatly separated from the support (Figure 7b). Moreover, the morphology of the particles is retained and the support is correctly segmented.

Figure 7c shows the particle size distribution (PSD) in terms of equivalent diameter (i.e., the diameter of the sphere whose volume equals that of the segmented particle). According to this PSD, the size of the Pd nanoparticles ranges between 0.5 and 4.3 nm, with an average size of 2.2 nm. The PSD obtained manually by conventional methods, i.e., by visual inspection of raw 2D HAADF-STEM images, is slightly shifted toward higher values, ranging from 1.7 to 4.8 nm, with an average value of 3.3 nm (Figure SI 8). As also shown in this Figure, the differences between both PSDs concentrate in the size region below 1.5 and above 4.0 nm.

The difference observed between both PSDs in the small size range can be explained if one takes into account that the smallest nanoparticles are those providing the lowest contrast in the experimental images, being, therefore, the most difficult to detect by the eye. In contrast, in the fully automated 3D analysis, each detected particle is the result of putting together the information of a large collection of images (29 images in the tilt series). Some of the 2D projections may not show the presence of some particular small particles due to contrast limitations, but they become visible in other projections of the set. Therefore, collecting a tilt series inherently increases the chances of detecting the smallest particles in an aggregate.

Regarding the difference in the size range of 4.0 nm and above, the two following factors should be considered. First, the natural bias in a manual procedure toward the measurement of larger nanoparticles, which are those showing the highest contrast. Second, the fact that the dimensional magnitude measurable in 2D HAADF-STEM images is the projected size of the particles. This magnitude appears adequate if the particles are isolated and mostly equiaxial. However, when individual small particles overlap in projection, not only a smaller number of small particles is detected, but also the diameter of an apparently large particle is mistakenly overestimated. Likewise, when the particles are not equiaxial, as is the case of some detected in our Pd/CeO_2_ catalyst, a diameter derived from the actual volume of the particle provides a more precise description of size.

In summary, the loss of information in the range of smallest nanoparticles, together with the possible bias and overestimation of particle size, could explain the difference between the PSDs established from ET and 2D HAADF-STEM images. In any case, the limitations of the 2D analysis in this type of complex systems should be highlighted since they affect, as shown below, the estimation of other important parameters, such as the dispersion of the supported phase.

Metal dispersion (*D*), a parameter that measures the ratio of surface atoms exposed at the surface of the whole nanoparticle system with respect to the total number of atoms in the catalyst, is generally used as a key parameter to understand the catalytic behavior of supported catalysts.^41^ Conventional measurements involve analyzing PSDs obtained by 2D STEM/TEM images or data obtained by macroscopic techniques based on chemisorption of small molecules such as H_2_ or CO. Regarding Pd, Gatica et al. have reported the limitations of metal dispersion determination in Pd nanocatalysts supported on ceria-based oxides due to (i) the poor contrast exhibited by the nanoparticles in TEM images and (ii) the formation of Pd hydride in H_2_ chemisorption experiments. The former contributes to underestimate dispersion since the contribution of the smallest particles is lost in the analysis, whereas the second one overestimates the dispersion value since bulk hydrogen species are attributed to adsorption by atoms exposed at the surface.^42^

The determination based on ET is clearly more reliable, as it involves a direct measurement of the two basic parameters required for its estimation, i.e., the surface (*S*_i_) and volume (*V*_i_) of individual nanoparticles. With this information, the dispersion of the supported phase is defined as follows
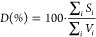

A dispersion of 63% was obtained from the PSD established from ET, which is a value significantly larger than that obtained from the PSD determined by visual inspection of 2D HAADF-STEM images, 38%. These results illustrate the large influence of the limited contrast of the metal on the structural analysis of light metal nanoparticles supported on heavy substrates.

Moving to a different question, Pd nanoparticle morphology was evaluated in terms of sphericity (ε). This parameter, which also depends on the surface and volume of the particle, intends to evaluate how much the morphology departs from that of a perfect sphere. According to its definition, a perfect sphere shows a value of ε = 1. Values lower than 1 indicate deviation from a perfect sphere. As a reference, the sphericity of a hemisphere equals 0.83.

Figure 7d displays the values of sphericity (ε) as a function of size for the whole set of particles segmented from the ET reconstruction. Note how the sphericity of most nanoparticles falls within the 0.8–1.0 range. Only a very limited number of nanoparticles, among the largest ones, display sphericity below 0.8. A similar behavior has been observed in reconstructions of other aggregates in this catalyst (Table SI 2).

To better understand the relationship between sphericity and particle size, the data points in the ε vs *d* plot were analyzed using K-means clustering. This algorithm is an unsupervised machine learning method that classifies data points into groups or clusters around the centroids that better represent the properties of each cluster. Thus, this analysis provides, in this particular case, a sphericity value for each representative particle size range.

Figure 7e plots the K-means cluster assignments and centroids of the data points. In particular, 4 clusters were found whose properties are listed in Table 1. Note how the sphericity decreases monotonically with particle size. For the smallest nanoparticles (centroid 1), with size below 1 nm, sphericity is very close to 1, whereas for larger ones, those in clusters 2–4, sphericity drops down to values around 0.9, in the order of those corresponding to faceted (polyhedral type) morphology (e.g., dodecahedron ε = 0.91), in good agreement with previous results; Figure 6b–d.

**Table 1:** Different Properties of the Centroids Obtained from K-Means Clustering

	centroid 1	centroid 2	centroid 3	centroid 4
particle size (nm)	0.80	2.99	1.48	2.17
aphericity (ε)	0.98	0.88	0.92	0.89
truncation (α)	0.31	0.82	0.67	0.79
contact angle (θ) (deg)	134	100	109	102

In a recent study of gold nanoparticles supported on ceria cubes, sphericity has been related to the extent of truncation (α) of a sphere along its diameter.^16^ In this work, the truncation of the sphere was parametrized in such a way that no truncation corresponded to α = 0, truncation to a hemisphere to α = 1, and total truncation to α = 2. With this parametrization, the truncation value of each representative cluster was determined; Figure 7f.

In particular, values below 1 were obtained; Table 1. These figures indicate that, in all cases, the truncation plane is located below the center of the sphere. Therefore, in general, particles contact the surface of the support at angles above 90°; Table 1. Moreover, since truncation increases monotonically with particle size, these results suggest that larger particles wet the surface more strongly than smaller ones.

It is known that the growth of nanoparticles is influenced by different parameters, such as size, the nature of the chemical gaseous environment surrounding the particle, and the exact structural nature of the nanoparticle||support interface.^43^ In a study of the contact of Pd and Pd–Au nanoparticles grown on TiO_2_ by pulsed laser deposition, Nguyen et al. reported that the incorporation of gold atoms into the Pd lattice induced a loss of adhesion, which was attributed to an increase in lattice strain.^44^ In our case, Pd nanoparticles locate preferentially onto the edges and corners of the nanocubes, i.e., onto {110} and {111} ceria facets. It can, therefore, be hypothesized that the increase of adhesion can be related to a decrease in total strain with size for Pd particles grown onto this type of facet. This would be in good agreement with previous works reporting a decrease in total compressive strain with size for Au nanoparticles grown onto {111} facets of fluorite-type ceria-based oxides.^45^

It can also reasonably be hypothesized that strain must be linked to the structural mismatch between the lattice parameters of the two phases. Such mismatch will depend, at least, on the crystallography of the planes contacting at the interface and the values of the lattice parameters of both phases.

Regarding lattice parameters, an increase of this magnitude with size has been reported for metal nanoparticles. In the case of Pd, Wang et al.^46^ have reported that such an increase is particularly steep in the size range observed in the catalyst studied in this paper (below 4 nm). Particularly, for Pd nanoparticles in the 2.5–3.5 nm size range, lattice parameter values 2% smaller than that of the bulk phase are observed, which is that expected for a perfect match between Pd and CeO_2_ in the growth relationship mentioned above. For smaller particles, the shrinkage of the Pd lattice becomes larger than this optimum value. This could contribute to an increasing strain for those smaller particles.

Likewise, it has also been reported that the value of metal nanoparticle surface tension increases with decreasing particle size.^47^ Since γ_Pd_ increases and cos(θ) becomes more negative with decreasing size, the Young–Dupre equation (eq 1 below) indicates that the interface energy becomes larger. This suggests that in the size range observed in the catalyst investigated in our paper, the metal–support interaction weakens with decreasing size.
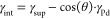
1

In summary, it has been possible to determine three main questions: (i) the PSD of the metal phase, (ii) the nature of the crystallographic planes onto which the metal particles grow, and (iii) the truncation degree of the metal nanoparticles. The first one determines the fraction of atoms exposed at the surface of the metal nanoparticles and the two latter basic aspects of the metal–support interface. As mentioned above, these features may have significant consequences on catalytic activity. In fact, several studies comparing the performance of Pd nanocatalysts supported on ceria cubes, rods, and octahedrons in various reactions have suggested that the catalytic activity of this system is highly influenced by the interaction between Pd and the support at interface sites.^48,49^ However, in none of these studies the actual crystallographic nature of the contacts at the metal||support interface is reliably backed up by 3D characterization data, as has been done here.

## Conclusions

Summarizing the whole set of results, it has been proved that the 3D analysis of ″light″ metal nanocatalysts, made up of small nanoparticles of low-atomic-number metals (3d, 4d) supported on crystallites of heavy oxides, by STEM ET is severely limited by both the contrast mechanisms prevailing in this type of systems and the deleterious influence of noise. A methodology has been developed that allows us to face this complex nanostructural characterization challenge and provide both qualitative and quantitative information about structural and morphological parameters. This methodology exploits the specific potential of different types of deep learning techniques and neural networks to optimize each stage of the electron tomography experiments, as well as the improved capabilities of advanced 3D reconstruction algorithms based on compressed sensing.

In particular, single-image super-resolution techniques based on deep learning have been used to remove noise in a smart way and enhance the quality of the HAADF-STEM tilt series. This implementation enables the visualization of even the smallest Pd nanoparticles in both profile and planar views, certainly a key aspect in the whole 3D analysis, which simply cannot be reached by means of conventional methods.

Likewise, image-restoration methods based on U-net generative adversarial network algorithms have also been used to correct artifacts linked to the alignment step. This has provided enhanced final reconstructions when compared to those obtained just after denoising the tilt series. These improvements have enabled us to determine, first, the qualitative properties of this type of nanocatalysts, specifically, the spatial distribution and general morphology of Pd nanoparticles. Results in this respect have revealed that the metal nanoparticles localize preferentially on the edges and corners of the nanocubes, i.e., grown on {110} and {111} facets. In good agreement with HR-HAADF and HR-BF images, nanoparticles depict a well-faceted morphology.

Finally, the 3D volumes obtained after the correction of misalignment effects were split into its components (metal and support) by means of semantic segmentation using a convolutional neural network based on U-net. This allowed a quantitative analysis, with statistical significance, of structural parameters related to dimensional aspects, such as particle size or metal dispersion, as well as of others related to quantitative aspects of morphology, like sphericity and truncation of the nanoparticle or contact angles at the metal||support interface.

Importantly, training of the different neural networks required creating large sets of computer-generated 3D models with structural and morphological characteristics resembling very closely those of the materials investigated. Though complementary, this is also a quite important outcome of this work.

This work faced a characterization challenge at the limits of complexity. Therefore, the methodology here developed clearly has the potential to be fruitfully applied to a wide range of metal catalysts, making it a versatile tool for the 3D analysis of complex systems. In particular, nanostructured catalysts based on other 4d elements such as Ru and Rh or 5d noble metals, like Pt, Au, or Ir, widely used in a variety of processes, fall completely within reach for this type of study. Moreover, the analysis of structural and morphological properties relevant to chemical or functional properties of other nanomaterials mixing a small-sized, light (low *Z*), component with a second, heavier one of larger dimensions will also benefit from the DL-CS methodology optimized in this work.

## Experimental Methods

### Sample Preparation

The catalyst prepared for this study was synthesized employing a two-step preparation process. The ceria oxide nanocubes, CeO_2_-NC, were obtained by a hydrothermal method following the recipe reported in reference.^34^

In a second step, the Pd/CeO_2_-NC catalyst was prepared via deposition–precipitation using PdCl_2_ (Sigma-Aldrich 99% purity) as palladium precursor, Na_2_CO_3_ (0.05M) as a precipitating agent, and 5 g of the support. The palladium precursor was added to the support suspended in an aqueous solution at pH = 8 and at 60 °C for 1 h, under stirring. Afterward, the suspension was further aged in solution under the same conditions for 1 h. The obtained precipitate was filtered and washed with deionized water several times to remove chlorides. Finally, the catalyst was dried at 100 °C and further pretreated in a 5% O_2_/He gas mixture at 250 °C for 1 h and subsequently another hour in He flow at the same temperature.

### Tomography Experiments

Electron tomography (ET) experiments were performed using an FEI Titan^3^ Themis 60–300 double aberration-corrected microscope operated at 200 kV. A convergence angle of 9.0 mrad was selected in order to improve the depth of focus. HAADF, DF4, and DF2 detectors were used. In particular, a camera length of 58 mm was used, providing a collection angle range of 91–198, 24–91, and 12–24 mrad, respectively. The series of STEM-HAADF/DF images (512 × 512 pixels) at different tilts were recorded manually using an acquisition time of 3.15 s/frame, a beam current of 50 pA, and a pixel size of 0.249 nm. This makes a total dose of roughly 600 e-/Å^2^ per image. Images were acquired at tilt angles, with respect to the incoming electron beam, within the −70 - + 70° range every 5° (i.e., at −70, −65, −60,······+70°), which makes a total of 29 images per tilt series.

The whole set of images was aligned by combining the cross-correlation method, using FEI Inspect3D v4.1, and the landmark-based alignment implemented in TomoJ v2.31. Afterward, they were reconstructed into a 3D volume using a compressed sensing algorithm based on the minimization of the total variation (TVM) of the individual HAADF-STEM images. In particular, a 3D implementation of the TVAL3 routine, using an ASTAR toolbox, was employed. For visualization and further nanometrological analysis of the reconstructed volumes, the FEI Avizo Software and homemade Matlab scripts were used.

### Deep Learning Implementation

Deep learning algorithms were implemented in TensorFlow and Keras in an Intel Core i9–10900 CPU@2.80GHz with a GPU Nvidia GeForce RTX 3090. To denoise the tilt series, an enhanced super-resolution generative adversarial network (ESRGAN) architecture was adapted for our problem. In our implementation, we made some modifications to the original architecture. Specifically, the dense block and a residual in residual dense block (RRDB) were modified to adequate them to our workstation system. Additionally, we employed RaGAN (relativistic generative adversarial network) as a cost function for both the generator and discriminator. For the training of the network, first, the generator (ESRResnet) was trained for 100 epochs, a batch size of 10, and using an Adam optimizer with a learning rate of 0.0001. Afterward, the generator and discriminator were trained for 100 epochs, a batch size of 5, and using an Adam optimizer with a learning rate of 0.0001. We followed this procedure to provide a first solution in order to optimize the process of training.

To perform image restoration, our adapted ESRGAN network was modified by implementing a U-net-based autoencoder architecture. This architecture is a 3 number of layers for both the encoder and decoder. For the encoder, each layer contains 2 convolution layers and 1 of average pooling, while in the decoder, 1 unsampled layer with subpixel convolution, followed by 2 convolution layers and an add layer, which are the skip connections. This configuration is that of the generator; the discriminator remained unchanged. We trained the models for 100 epochs, using a batch size of 15 for the generator and 8 for the generator and discriminator. An Adam optimizer with a learning rate of 0.0001 was used. A scheme showing a diagram of this deep learning architecture is included in the Supporting Information.

Finally, for segmentation, a residual U-net structure using an AttenResUnet model was implemented. To train this model, 30 epochs were used with a batch size of 25, an Adam optimizer with a learning rate of 0.001, and a Binary Focal Loss was used as the loss function to avoid the disparity of classes.

## Supporting Information Available

Pd_DeepLearning_SI.pdf. This file contains supporting data related to building material-realistic 3D models of Pd nanoparticles supported on CeO_2_ nanocubes; simulating images to train the different deep learning neural networks (DL-NN); details of the DL-NN architecture used for image restoration; experimental 2D HR-HAADF and HR-BF STEM images of a 1.2% wt Pd/CeO_2_-NC catalyst; and particle size distribution obtained from the manual analysis of 2D HAADF-STEM images of the 1.2% wt Pd/CeO_2_-NC catalyst (PDF)

## Abbreviations

ET: electron tomography
3D: three dimensions
HAADF: high-angle annular dark field
STEM: scanning transmission electron microscopy
CS: compressed sensing
SIRT: simultaneous iterative reconstruction technique
WBP: weighted back projection
TVM: total variation minimization
DL: deep learning
EDX: energy-dispersive X-ray spectroscopy
GAN: generative adversarial network
CNN: convolutional neural network
SR: super resolution
SEM FIB: scanning electron microscopy focused ion beam
ESRGAN: enhanced super-resolution generative adversarial network
PSNR: peak signal-to-noise ratio
SSIM: structural similarity index measure
AT-UWT: Anscombe and undecimated wavelet transforms
RIDnet: residual Image denoising with feature attenuation
DP: deposition–precipitation
TEM: transmission electron microscopy
