# Supplementary material for: Quantitative 3D Characterization of Functionally Relevant Parameters in Heavy-Oxide-Supported 4d Metal Nanocatalysts
Source: Chem Mater. 2023 Sep 14;35(18):7564–76. doi: 10.1021/acs.chemmater.3c01163 (PMC10538501; doi:10.1021/acs.chemmater.3c01163)
Supplement: Supplementary file 1 — cm3c01163_si_001.pdf [file cm3c01163_si_001.pdf]

Supporting Information

Quantitative 3D Characterization of Functionally-relevant parameters in Heavy-Oxide Supported 4d Metal Nanocatalysts

José Marqueses-Rodríguez, Ramón Manzorro, Justyna Grzonka, Antonio Jesús Jiménez-Benítez, Lionel Cervera Gontard, Ana Belén Hungría, José Juan Calvino, Miguel López-Haro **

Departamento de Ciencias de los Materiales e Ingeniería Metalúrgica y Química Inorgánica. Facultad de Ciencias. Campus Rio San Pedro S/N. Puerto Real. 11510. Cádiz. Spain.

Image Quality Metrics

PSNR is defined [1] as:

$$PSNR = 10 \cdot \log_{10}\left(\frac{MAX_I^2}{MSE}\right) = 20 \cdot \log_{10}\left(\frac{MAX_I}{\sqrt{MSE}}\right) \qquad MSE = \frac{1}{MN}\sum_{i=0}^{M-1}\sum_{j=0}^{N-1}||I(i,j) - K(i,j)||^2$$

Where $MAX_I=2^B-1$, with B the number of bits used for the representation of image intensity values (dynamic range). Note that MSE (Mean-Squared Error) measures the average quadratic deviation between the two digital, MxN, images which are compared. In our case I(x,y) would correspond to the noise-free image whereas K(x,y) would correspond to the image obtained after denoising.

Therefore, PSNR quantifies, in decibels, how large is the maximum signal to MSE ratio. Typical values fall in the 20-40 dB range, the quality of the denoising method increasing with PSNR.

The Structural SIMilarity (SSIM) index [2] has been used to calculate the agreement between the reconstructions and the original synthetic models. This index takes values between 0 and 1; the higher the values the more similar the images, where a value 1 means that both images can be considered identical. SSIM index takes into account for the comparison three characteristics of the images: luminance, contrast and structure.

[1] A.N. Netravali and B.G. Haskell, Digital Pictures: Representation, Compression, and Standards (2nd Ed), Plenum Press, New York, NY (1995).

[2] Z. Wang, A. C. Bovik, H. R. Sheikh, and E. P. Simoncelli, "Image quality assessment: From error visibility to structural similarity." IEEE Transactions on Image Processing, vol. 13, no. 4, pp. 600–612, 2004.

Additional Figures & Tables

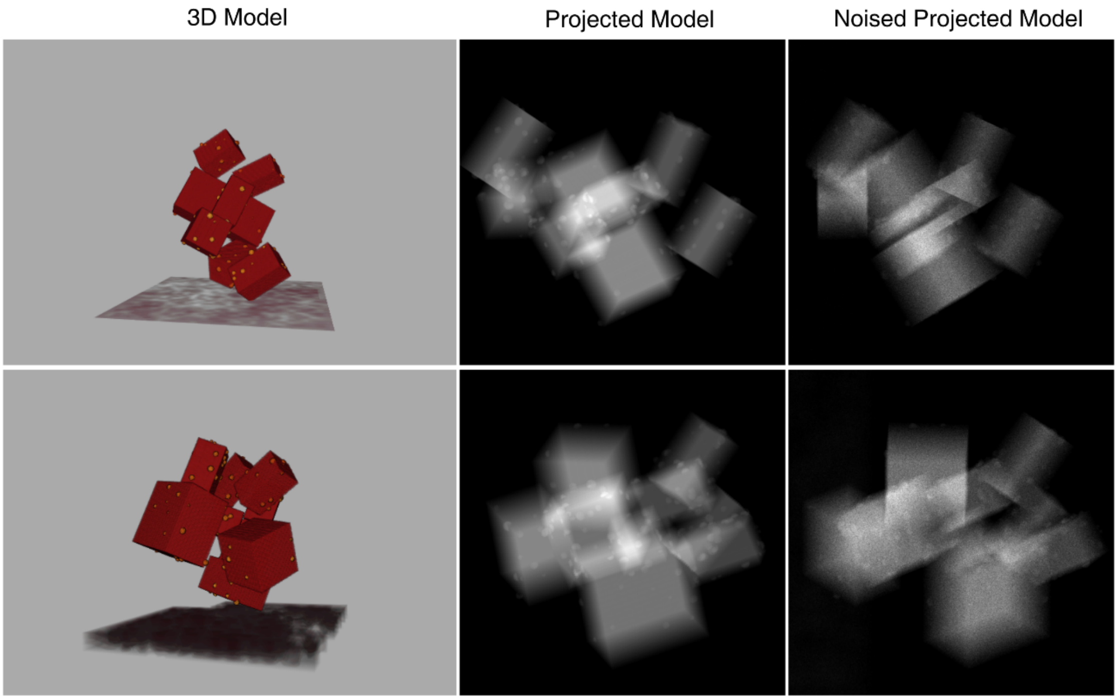

Figure SI 1. (left) Perspective views of 3D computer-generated models; (middle), simulated projected images after tilting 10º; (right) simulated projected image after adding a mixture of Poisson and Gaussian noise.

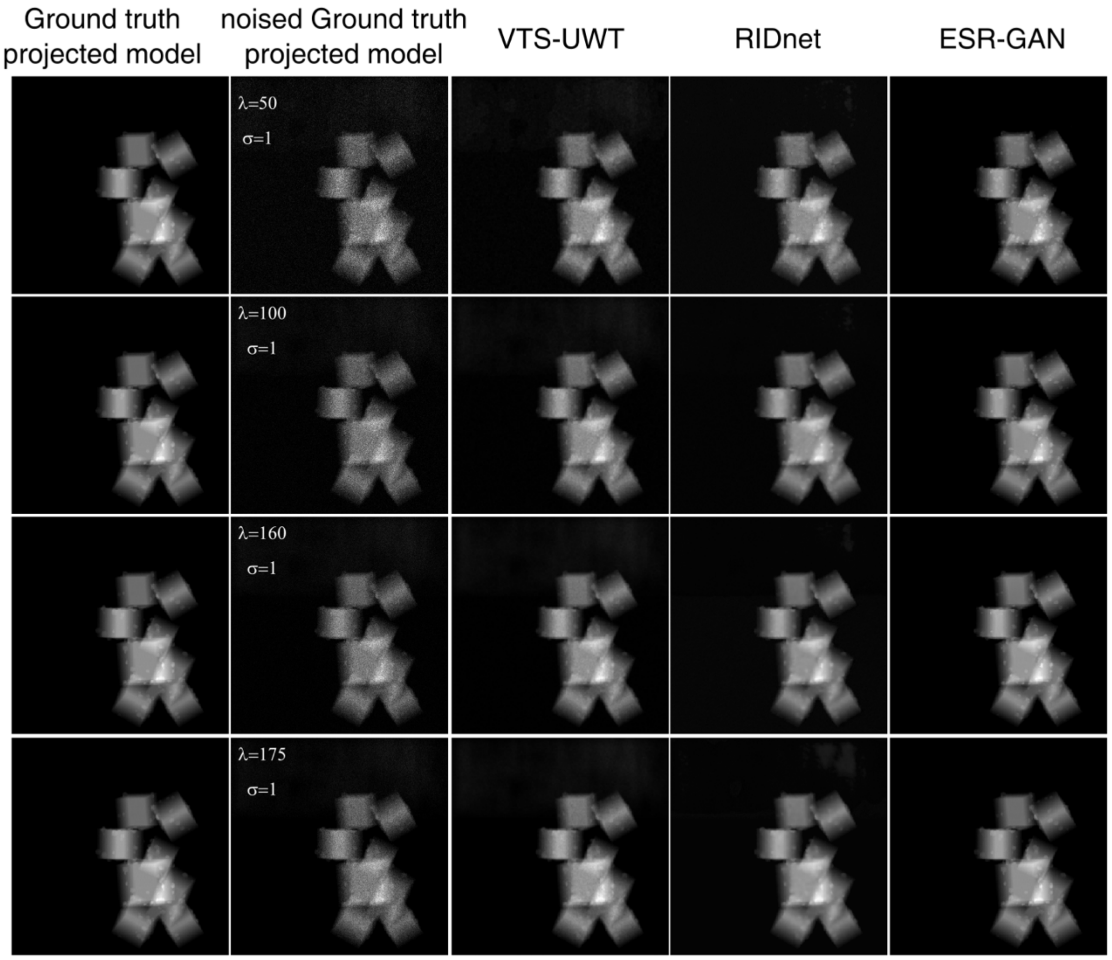

Figure SI 2.- (first, leftmost column) Ground truth projected model; (second column) noised Ground truth projected model after adding different levels of poisson (λ) and Gaussian (σ) noise; (third column) after denoising using VST-UWT; (fourth column) after denoising using RIDnet; (fifth column) after denoising using ESR-GAN.

Training Data for image restoration

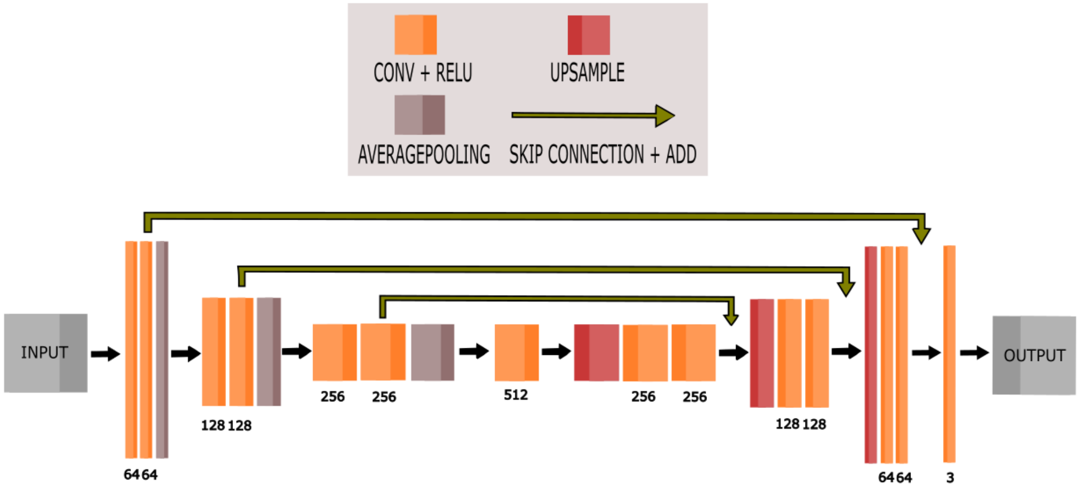

Figure SI 3.- Schematic illustration of the U-net based auto-encoder architecture of our adapted ESRGAN network.

This step becomes crucial when we are dealing with data in which an external reference is required, as it is the case of microscopy and more particularly of HAADF-STEM ET.

To train the U-net GAN structure, both G and D were trained. For G the synthetic models, denoised using the ESRGAN network, were reconstructed using the CS-TVM3D algorithm, Figure SI 4b. Then each slice was on-purpose misaligned in both shift and tilt axis rotation to reproduce the so-called "arc" effect.

For D, the perfect 3D synthetic models, without any distortion, were used as input, Figure SI 4a. The latter were used as the ground truth which the D uses to provide the correct answer to G. This training provides a final prediction, Figure SI 4c. SSIM values close to 1 were in general obtained.

Around 6000 images have been used for this training. In fact, 80% of these images were used for training and 20% to validate the predictions.

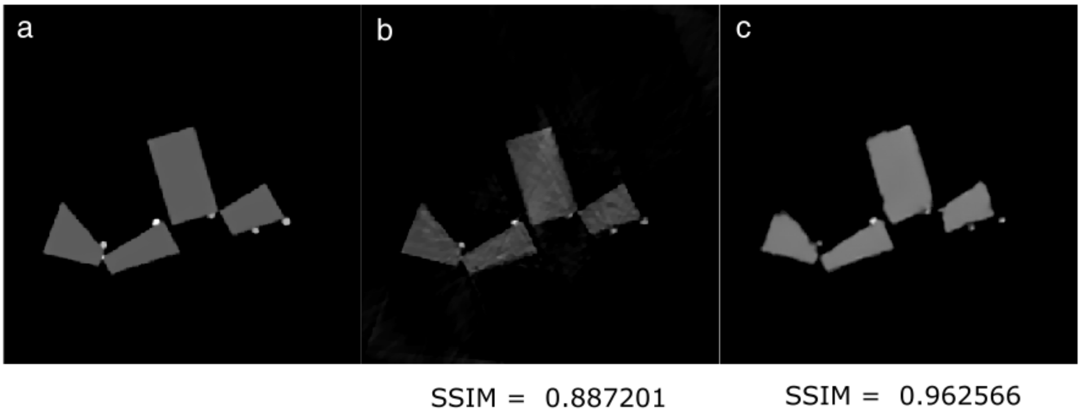

Figure SI 4.- A representative slice of one of the synthetic models: (a) without any distortion. Ground truth; (b) the reconstructed slice without correction of misalignment (c) the reconstructed slice after misalignment correction.

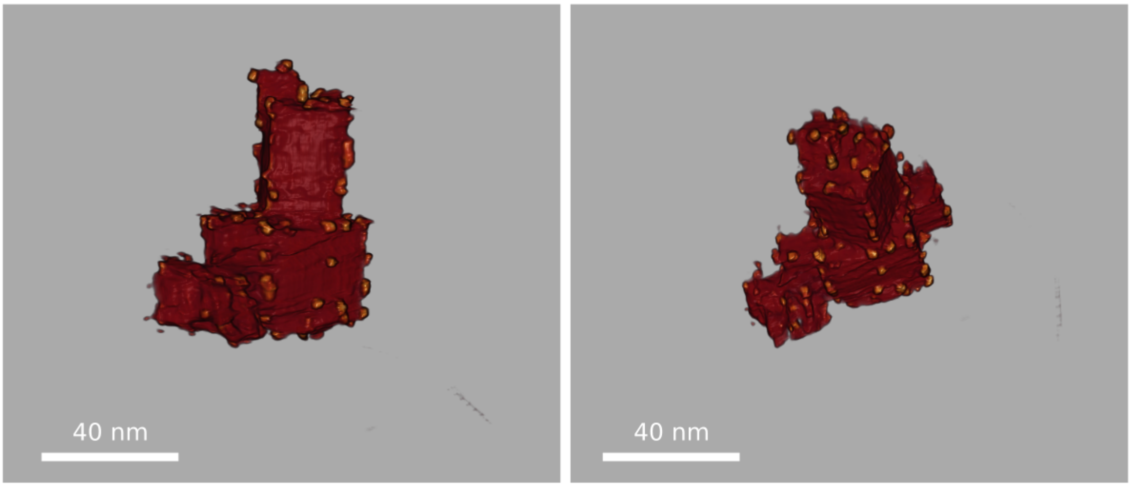

Figure SI 5.- Perspective views of the 3D reconstruction of an aggregate of the Pd/CeO_2 catalyst.

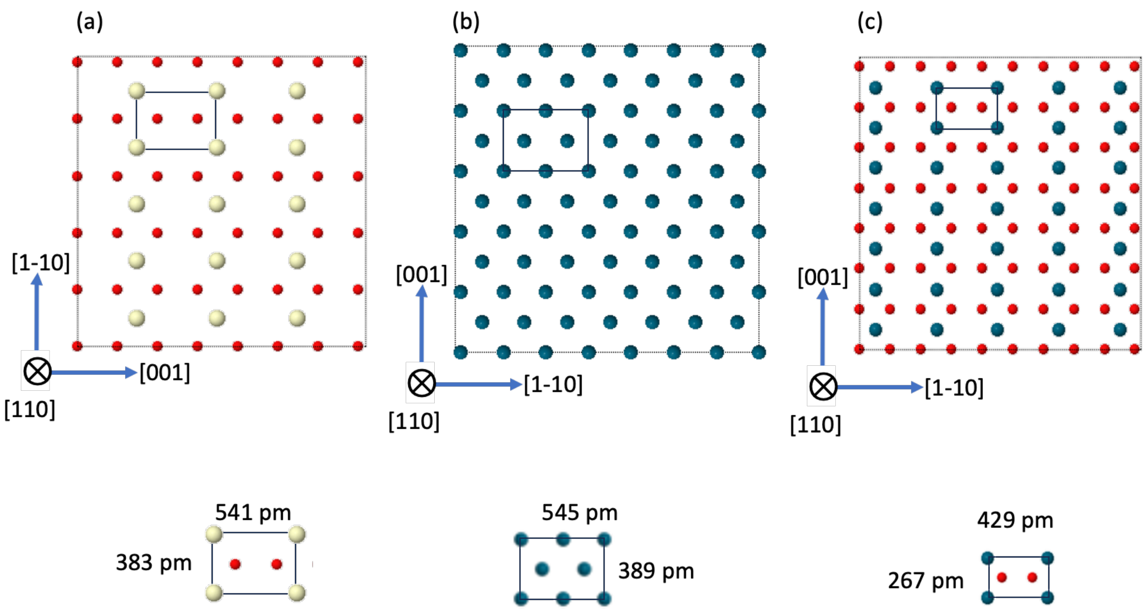

Figure SI 6.- Structural models of the {110} planes of: (a) CeO_2; (b) Pd and (c) PdO.

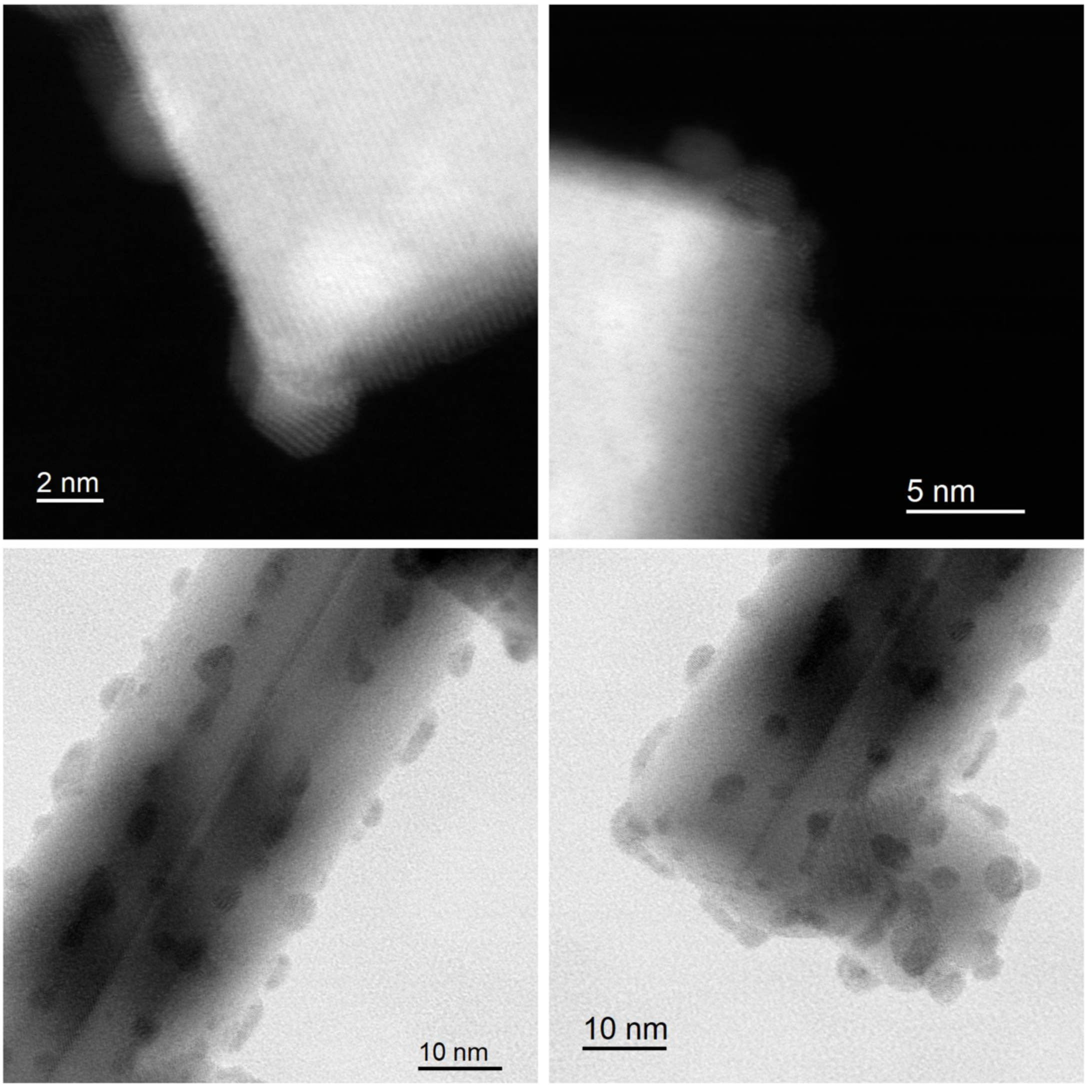

Figure SI 7.- Representative HR-HAADF (top) and HR BF-STEM images (bottom) of Pd nanoparticles supported on CeO_2 NC.

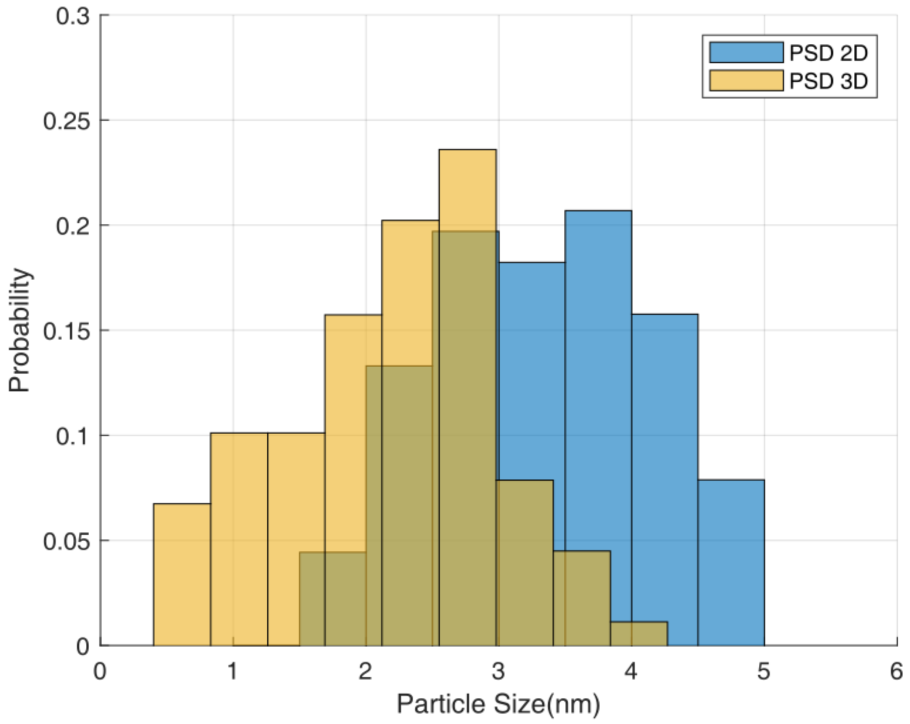

Figure SI 8.- Comparison of the PSDs obtained from the tomographic 3D (bars in blue) and conventional 2D (bars in red) analysis.

Table SI-1. Estimation of PSNR and SSIM values for noised and denoised models using VTS-UWT, RIDnet and ESRGAN algorithms.

	Noised Phantom		VTS UWT		RIDnet		ESR-Gan	
	PSNR	SSIM	PSNR	SSIM	PSNR	SSIM	PSNR	SSIM
λ=50 σ=1	24.61	0.13	32.33	0.34	29.81	0.27	38.70	0.97
λ =100 σ =1	27.67	0.27	34.53	0.49	33.49	0.36	40.30	0.99
λ =160 σ =1	29.2	0.39	35.39	0.58	32.58	0.35	40.83	0.99
λ =175 σ =1	30.28	0.62	36.31	0.69	31.96	0.38	40.74	0.99

Table SI 2. Different properties of the centroids obtained from K-means clustering obtained from the analysis of the aggregate shown in Figure SI 5.

	Centroid 1	Centroid 2	Centroid 3	Centroid 4
Particle size (nm)	0.95	2.54	1.80	3.36
Sphericity (ε)	0.95	0.85	0.88	0.81
Truncation (α)	0.51	0.94	0.84	1.08
Contact angle (θ) (deg)	119.3	93.4	99.2	85.4
